# Supplementary material for: Complexome profiling of the Chlamydomonas psb28 mutant reveals TEF5 as an early PSII assembly factor
Source: Plant Cell. 2025 Mar 17;37(6):koaf055. doi: 10.1093/plcell/koaf055 (PMC12164590; doi:10.1093/plcell/koaf055)
Supplement: koaf055_Supplementary_Data [file koaf055_supplementary_data.zip › psb28_interactive.zip!/plots/intensity/qualityPlots/peptides/_Cre01.g037150.t1.1.html]

### Cre01.g037150.t1.1

Trivial name:   
SeqCoverage [median]: 8.00  
Mass: 96.13 kDa  
  
 

| Sequence | MissedCleavage | Mass [Da] | wtIntensity | muIntensity | Score |
| --- | --- | --- | --- | --- | --- |
| AMEGVTDDSAK | 0 | 1122.49 | 315.00 | 411.89 | 68.89 |
| SASVRTTSVSPGR | 1 | 1303.68 | 0.00 | 7225.87 | 42.09 |
| SPDPAAAAAAANAR | 0 | 1252.62 | 388.88 | 0.00 | 107.79 |
| TQGQALGIAGAGTHLVGR | 0 | 1705.92 | 0.00 | 484.83 | 41.86 |
| TVEDVLGTR | 0 | 988.52 | 689.35 | 2453.14 | 76.68 |
|  |  |  | 1393.2 | 10575.7 |  |
